# Discussion on Syphilis

**Published:** 1892-01

**Authors:** 


### DISCUSSION ON DR. WELLER'S PAPER ON SYPHILLIS.

Dr. Richmond (W. T., Manor, Texas), took the negative and argued against the transmissibility of syphilis directly from father to child. If the spermatozoa are effected with the syphilitic poison they would be blasted. Experiments have failed to produce syphilis by inoculation. The semen contains no syphilis, neither does the saliva. And a mother cannot catch syphilis from a syphilitic child nursing. I do not see how a mother can contract syphilis from the child in utero. If a mother contract syphilis she shows some evidence of it; few escape some evidence. Key relates a case of a child with syphilis who nursed a wet nurse and a chancre appeared on her nipple—the child's father had chancre, the mother was declared healthy; but syphilitic patches were found later. A year later came another syphilitic child. Mother apparently healthy, and declared she was. Subsequently she bore a healthy child. How did this mother acquire syphilis? Apparently from her husband. Dr. Richmond read a case from a book, [author not remembered], in which a man with syphilis came for treatment, bringing with him a woman also with syphilis. He had seduced her under promise of marriage. They were treated, successfully it seemed, and the marriage never took place. The woman became a belle in society, and after a while married another man. She had a syphilitic child. She might have nursed the child and caught it. She would never admit she had ever had the disease. Women never will own up; men will. So here appears a case where both parents werc (apparently) healthy, yet had a syphilitic child. Another woman, after two miscarriages, gave birth to a sound child. Father acknowledged that he had had syphilis; but at time of birth neither parent had any sign of the disease. So it seems mothers do have healthy children by syphilitic fathers. A mother under treatment may have a child born healthy and she may have a syphilitic child later. Key reports a case of the kind.

Prof. J. E. Thompson (Med. Dept. University of Texas), said: In discussing this question I would first mention certain arguments against it:

1. The fact that many fathers who are suffering from syphilis have children who are perfectly healthy.

This is granted in many cases, but one case which proves the contrary, is worth all the other cases of this nature; and cases of syphilitic fathers with syphilitic children are very common.

2. The fact that inoculation of the semen of syphilitic men is powerless to produce a syphilitic infection.

This is also true, but can we strictly compare fecundation with inoculation?

Fecundation is a process as mysterious as beautiful. To the ovum it gives life, and endows it with physiological and pathological attributes. It can transmit to the child certain physical properties, such as color of eyes, shape of eye-brow, tricks of movement and various other qualities; also, certain moral characteristics, which traits, both physical and moral are often the outcome of a generation previous. This being so it is quite erroneous to compare the two processes.

There are certain arguments in favor of this process:

1. The fact that syphilitic fathers can and do produce syphilitic infants, the mother remaining (apparently) healthy.

2. The fact that in households where the mother is (apparently) healthy and the father affected with syphilis, abortions and premature labors are excessively common.

3. The influence of treatment on the progress of pregnancies where the father is affected and the mother (apparently) healthy, showing that when the father alone is put under treatment, labor resulted at full term and the issue a healthy infant.

4. The causation of the so-called "syphillis par conception" the history is usually the following: A man, who has previously had syphilis, and who at the time of marriage does not show the slightest manifestations, marries a perfectly healthy woman. About the third or fourth month after marriage the woman becomes the possessor of a typical secondary syphilis. On careful examination no primary lesion whatever can be discovered and no bubo in the groins. The man also shows no manifestation on his person capable of infecting his wife, and denies ever having had any such manifestation since his marriage.

In these cases one considers that the woman has been infected with syphilis from the fœtus by the placental circulation.

Returning to the women, who, while apparently healthy, still bear syphilitic children, numerous observations have been made. Neuman tried to inoculate such women with syphilitic virus but failed. It has also been found that these women can nurse their syphilitic offspring with impunity. From these facts Fournier argues that they have received syphilis gradually from the fœtus by a slow process of inoculation through the placental blood and have so gained immunity.

---

[NOTE.—It is to be regretted that the JOURNAL was not able to get a full report of the discussion on syphilis.—ED.]

